# Insights into the biochemical defence and methylation of the solitary bee *Osmia rufa* L: A foundation for examining eusociality development

**DOI:** 10.1371/journal.pone.0176539

**Published:** 2017-04-27

**Authors:** Aneta Strachecka, Jacek Chobotow, Jerzy Paleolog, Aleksandra Łoś, Michał Schulz, Dariusz Teper, Halina Kucharczyk, Maciej Grzybek

**Affiliations:** 1 Department of Biological Basis of Animal Production, Faculty of Biology, Animal Sciences and Bioeconomy, University of Life Sciences in Lublin, Akademicka 13, Lublin, Poland; 2 Zoological Museum/Laboratory, Institute of Biology and Biochemistry, Faculty of Biology and Biotechnology, Maria Curie-Sklodowska University, Akademicka 19, Lublin, Poland; 3 Department of Zoology, Animal Ecology & Wildlife Management, Faculty of Biology, Animal Sciences and Bioeconomy, University of Life Sciences in Lublin, Akademicka 13, Lublin, Poland; 4 Research Institute of Horticulture, Apicultural Division in Pulawy, Kazimierska 2, Pulawy, Poland; 5 Department of Zoology, Institute of Biology and Biochemistry, Faculty of Biology and Biotechnology, Maria Curie-Sklodowska University, Akademicka 19, Lublin, Poland; 6 Department of Parasitology and Invasive Diseases, Faculty of Veterinary Medicine University of Life Sciences in Lublin, Akademicka 12, Lublin, Poland; 7 Department of Molecular Biology, Institute of Genetics and Animal Breeding, Polish Academy of Science in Jastrzebiec, Postepu 36A, Magdalenka, Poland; USDA Agricultural Research Service, UNITED STATES

## Abstract

We examined age-related biochemical and histological changes in the fat bodies and hemolymph of *Osmia rufa* males and females. We analysed solitary bees during diapause, in October and in April; as well as the flying insects following diapause, in May and June. The trophocyte sizes, as well as the numbers of lipid droplets were the greatest at the beginning of diapause. Subsequently, they decreased along with age. Triglyceride and glucose concentrations systematically decreased in fat body cells but increased in the hemolymph from October to June. Concentrations/activities of (enzymatic and non-enzymatic) antioxidant and proteolytic systems, as well as phenoloxidase, aspartate aminotransferase, alanine aminotransferase and alkaline phosphatase levels were constant during the diapause, usually lower in the males than the females. Prior to the diapause/overwintering, the concentrations/activities of all the compounds were higher in the fat bodies than in the hemolymph. Later in the spring and in the summer, they increased in the hemolymph and on the body surfaces, while decreasing in the fat bodies. The global DNA methylation levels increased with age. Higher levels were always observed in the males than in the females. The study will promote better understanding of bee evolution and will be useful for the protection and management of solitary bees, with benefits to the environment and agriculture.

## Introduction

There are two reasons to study age-related biochemical and histological changes in *Osmia rufa* L. (*O*. *bicornis;* red mason bee), particularly its biochemical defence characteristics.

**(1)** The appearance of insect eusociality has been one of the major transitions in evolution [1]. Although the selection mechanisms of this process are relatively well understood [2,3], biochemical mechanisms/consequences of apian eusociality remain obscure [4–6]. The eusocial bee female castes (queens and workers) and males (drones) exhibit different physiological bases of life span, resistance, senescence, etc. [7]. The division of labor among the workers, manifested in the presence of different worker age-castes (i.e. the age-polyethism, also present in winter workers), has to have broader biochemical consequences than just differences in main hormone (eg. JH) activities. Studies have been undertaken to investigate compounds which are elements of biochemical defence [8]. We believe, however, that in order to fully understand the biochemical evolution of eusociality, it is necessary to compare age-related biochemical changes in eusocial and solitary bees. Unfortunately, there is a significant gap in the knowledge about age-related biochemical processes in solitary bees, hindering such comparisons, which we decided to fill with this work. We used the solitary *O*. *rufa* bee, which only has fertile females and males differing in body size and physiology. Neither age polyethism nor female castes are present in it. Males and females at the diapause stage after the start of overwintering (in October), those just after the diapause/overwintering and at the beginning of the active life stage (in April) and flying, fully active insects (males and females in May, and only females in June) were collected for our analyses.

Two contrasting opinions predict the evolutionary consequences of sociality for immune systems. The first, sociality leads to stronger individual immunity because of the higher risk of disease transmission within crowded nests of social species. The second claims social species have evolved behavioral resistance that lowers disease risk within the group and results in lower immunity at the individual level [9–11]. However, we wonder which of these opinions may be applied to biochemical defence. To explain these controversies it is necessary to provide new information about this type of defence in solitary bees.

**(2)** As one of the main wild-living pollinators, *O*. *rufa* provides services for natural and rural ecosystems and is also employed in commercial pollination [12–16]. Protection of pollinating entomofauna is nowadays a major challenge. This is because of a harmful pressure of the current environment [17–19]. In this regard, it is particularly important to study the mechanisms of biochemical defense in *O*. *rufa* both for its preservation and commercial rearing.

### The biochemical bee defence

For understanding the biochemical bee defence it is crucial to determine the activities of the cuticular proteolytic system, which is the first anti-pathogen barrier, as well as the activities of the hemolymph proteolytic and antioxidative systems [20,21]. The hemolymph antioxidative system protects against reactive oxygen species (ROS). Proteins may undergo scission reactions with certain radicals/oxidants, leading to the direct formation of potentially toxic peptide fragments [22,23]. Various intercellular proteolytic enzymes in the hemolymph can recognize and preferentially degrade oxidatively damaged proteins to amino acids. Moreover, proteolytic system activates zymogenes, receptors and proteins from their precursors, degrading pathogen proteins at the same time [20,24,25]. In *O*. *rufa*, however, proteolytic and antioxidative activities were studied only in homogenates made of the entire individual bodies and only at the diapause stage [8,16,26,27] but never in mature, flying, fully active insects that are more exposed to environmental pressure (e.g. pesticides, ultraviolet radiation, ultrasound waves, toxic substances and pathogens). Therefore, our study concentrated on a comparative analysis of cuticle and hemolymph proteolysis in adult males and females.

### Additional biomarkers. Age-related changes

Biomarkers of the *O*. *rufa* biochemical defense, such as aspartate aminotransferase, alanine aminotransferase, alkaline phosphatase and phenoloxidase (PO), have been examined in this study. This has never been performed to date. These compounds support the functioning of the proteolytic and antioxidative systems in *A*. *mellifera* [21,28,29]. Moreover, PO is one of the oxidases responsible for the main part of oxygen uptake during the initiation of immune responses, as well as for morphogenetic processes in insects [30]. Therefore, it can be treated as a bee biochemical-defence and ontogenetic marker.

### Histological changes in the fat body

All the compounds involved in the biochemical defence (mentioned above) are synthesized in apian fat bodies (*corpus adiposum*), which function as metabolic centers. Moreover, fat bodies accumulate toxic and reserve compounds [e.g. urea, uric acid; [31]] and protect against oxidative stress, enhancing the apian resistance systems, as well as having effect on overwintering and post-wintering performance [32–35]. They actively participate in vitellogenesis [36–38]. There is a constant exchange between the fat body and the hemolymph with alternate phases of release and absorption of proteins [31,39]. Consequently, the fat body is crucial for biochemical defence mechanisms in bees. Therefore, we decided to apply an original approach in this study, involving the simultaneous analyzing of fat body cell structures and its biochemical characteristics, as well as the biochemical parameters of hemolymph. Moreover, fat bodies in *O*. *rufa* are almost unexplored, in particular their morphology and histology. We suppose that, the fat body structure of *O*. *rufa* (as a solitary bee) may be quite primitive and its exploration may help to explain how it could evolve to the more complicated tissue in eusocial insects. This will also help to explain the histological differences between apian workers and queens and answer the question whether the *O*. *rufa* female is more similar to honeybee workers or queens.

### Total DNA methylation

The functions and sequences of consistently methylated genes appear to have been especially well conserved over hundreds of millions of years of insect evolution [40,41]. Bonasio et al. [42] suggested that lower DNA methylation levels may be associated with the primitively eusocial and/or solitary bees in comparison to highly eusocial lifestyle. Global DNA methylation levels in eusocial bees, e.g. *A*. *mellifera*, increase with age and are also dependent on castes [43–45]. Changes in the global DNA methylation level are crucial for the response of the organism to environmental pressure [43]. However, we have found no information about global DNA methylation levels in *O*. *rufa*. Therefore, our research focused on the global DNA methylation analysis.

### Hypothesis and aim of the study

We decided to test the following hypotheses: (1) histological/morphological patterns of the fat body cells differ between the *O*. *rufa* males and females, and are dependent on the age; (2) biochemical defence characteristics such as the activities/concentrations of antioxidants, proteases, protease inhibitors, PO and some crucial enzymatic and non-enzymatic biomarkers in the *O*. *rufa* fat bodies and hemolymph are different between males and females; (3) values of all these parameters are decreasing with age; (4) the age and sex can influence not only the biochemical parameters in *O*. *rufa* (in a similar way to *A*. *mellifera*) but also global DNA methylation changes. The methylation level should be different than that reported for the social bee, *A*. *mellifera*.

Beyond checking hypotheses 1, 2, 3 and 4, the aim of this work was to provide a rich set of new, unpublished data on histology, biochemistry and DNA methylation in *O*. *rufa* that will be helpful for developing eusocial evolution theories and useful for the protection and management of solitary bees with benefits to the environment and agriculture.

## Results

### Histological changes in the fat body of *O*. *rufa*

**Fig 1:**
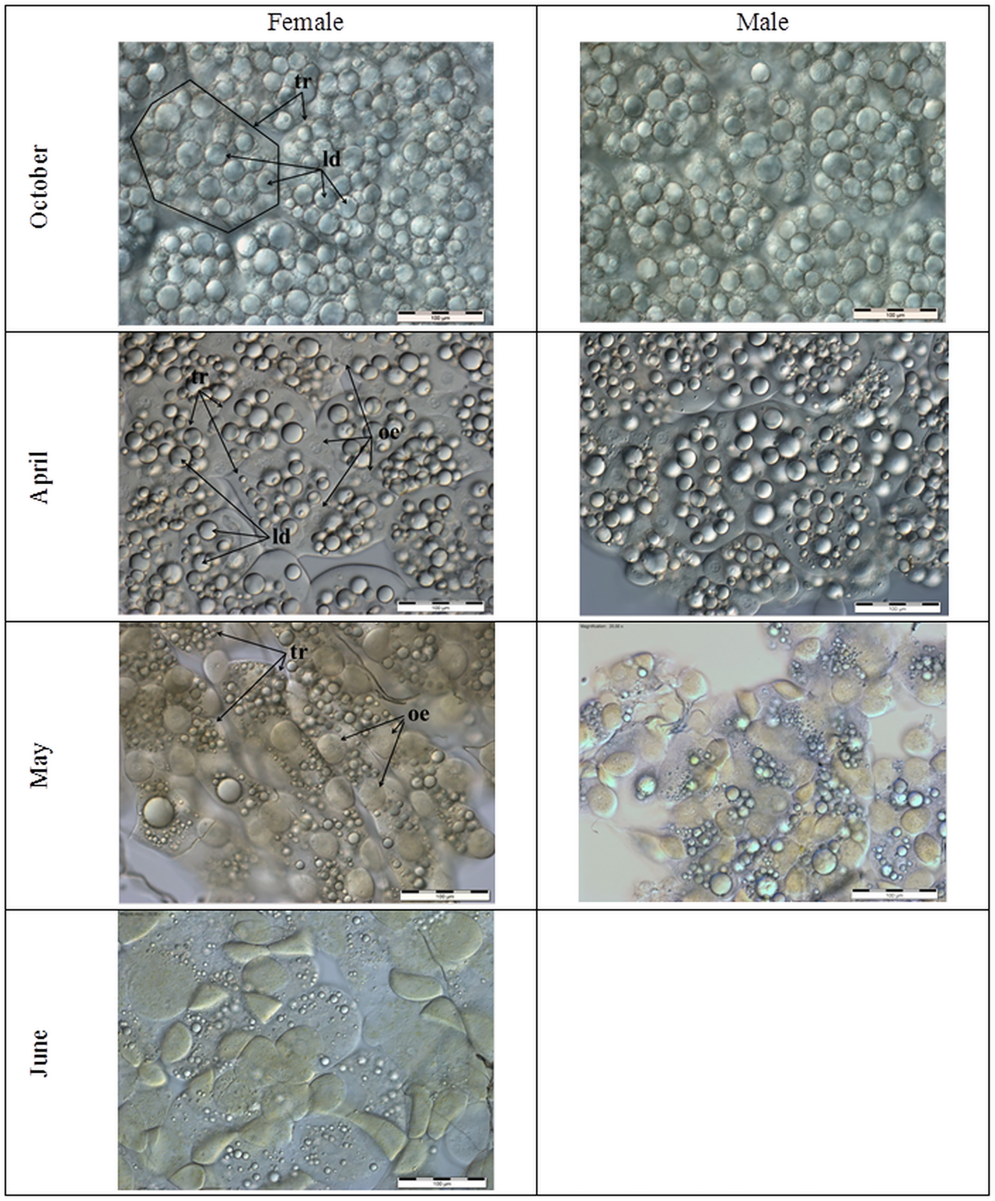
Histological features of fat body cells in *Osmia rufa* females and males. Scale bar– 100μm. Only females were analysed in June because males were not found. tr–trophocytes; oe–oenocytes; ld–lipid droplets.

Microscopic images of the fat bodies were similar in both sexes of *O*. *rufa* in October and April (Fig 1). Trophocytes were the only visible cells. Numerous lipid droplets filled entire trophocyte cells. These droplets were the largest at the beginning of the diapause/overwintering (in October). They decreased at the end of the diapause stage (in April). After the diapause/overwintering, the trophocyte sizes and volumes, as well as the numbers of the lipid droplets, dramatically decreased with age between April and June. In May, the fat body began to differ between the females and males. The males had fewer lipid droplets in trophocytes than the females. In April, oenocytes appeared among trophocytes in both sexes. They were scattered and had large nuclei containing granularities. However, the female oenocytes were oval and had poorly visible granularities in the cytoplasm, whereas they were of various shapes and had explicit granularities in the cytoplasm in the males. Intercellular spaces were visible between the fat body cells in both sexes. The numbers and sizes of the lipid droplets were lower in the females in June than in the previous months. The oenocytes changed shape from oval to polygonal and developed yellow granularities in the cytoplasm in this period.

### Biochemical changes in the fat body

**Fig 2:**
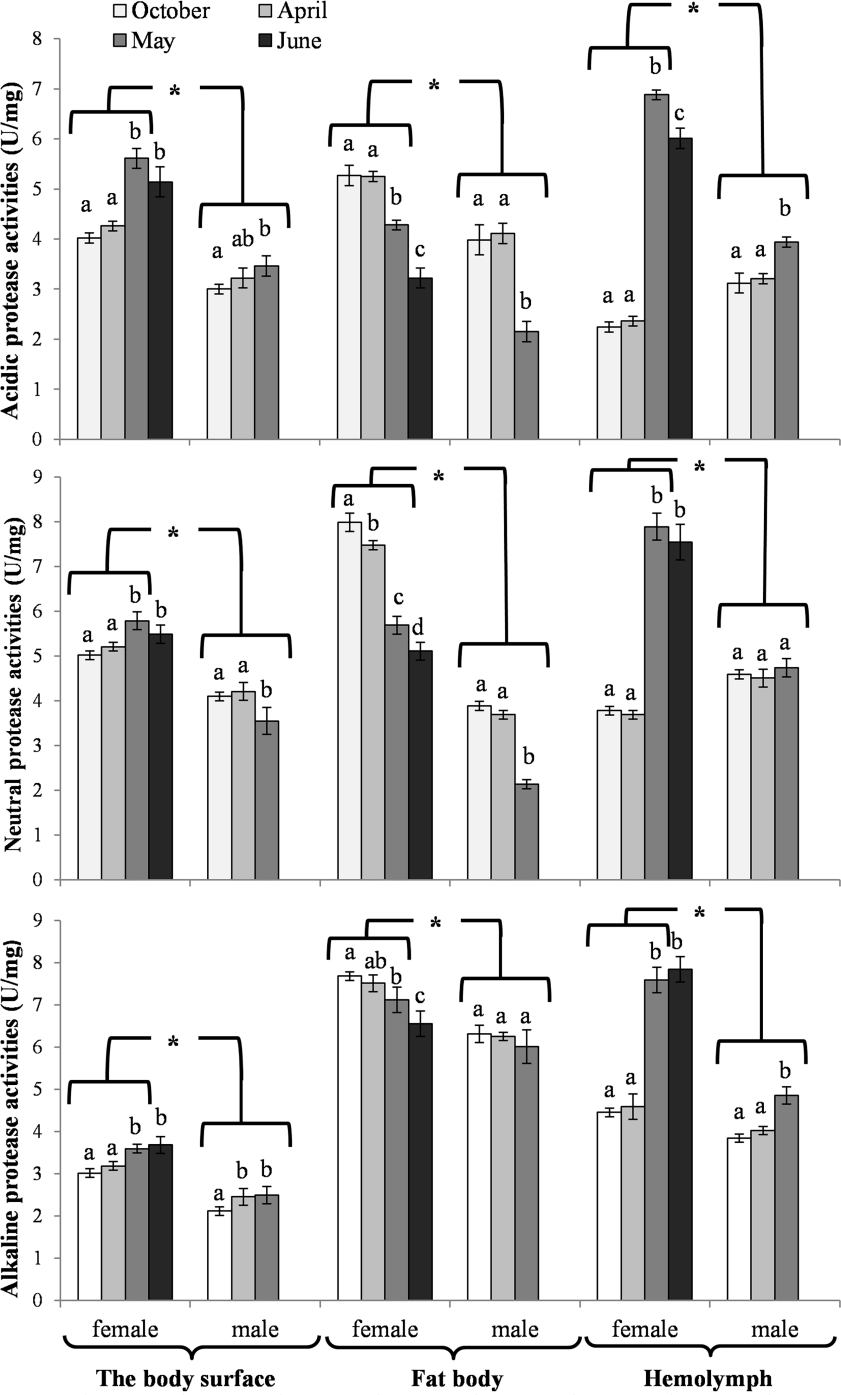
Acidic, neutral and alkaline protease activities on the cuticles and also in the fat bodies and hemolymph in *Osmia rufa*. Various lowercase letters indicate that the month averages differ significantly for comparisons made within each sex and tissue at P ≤ 0.05. The *asterisks* indicate significant differences (P ≤ 0.01) difference between sexes compared for the each month separately. The comparison was made separately within each of the tissue. Males were not found in June.

**Fig 3:**
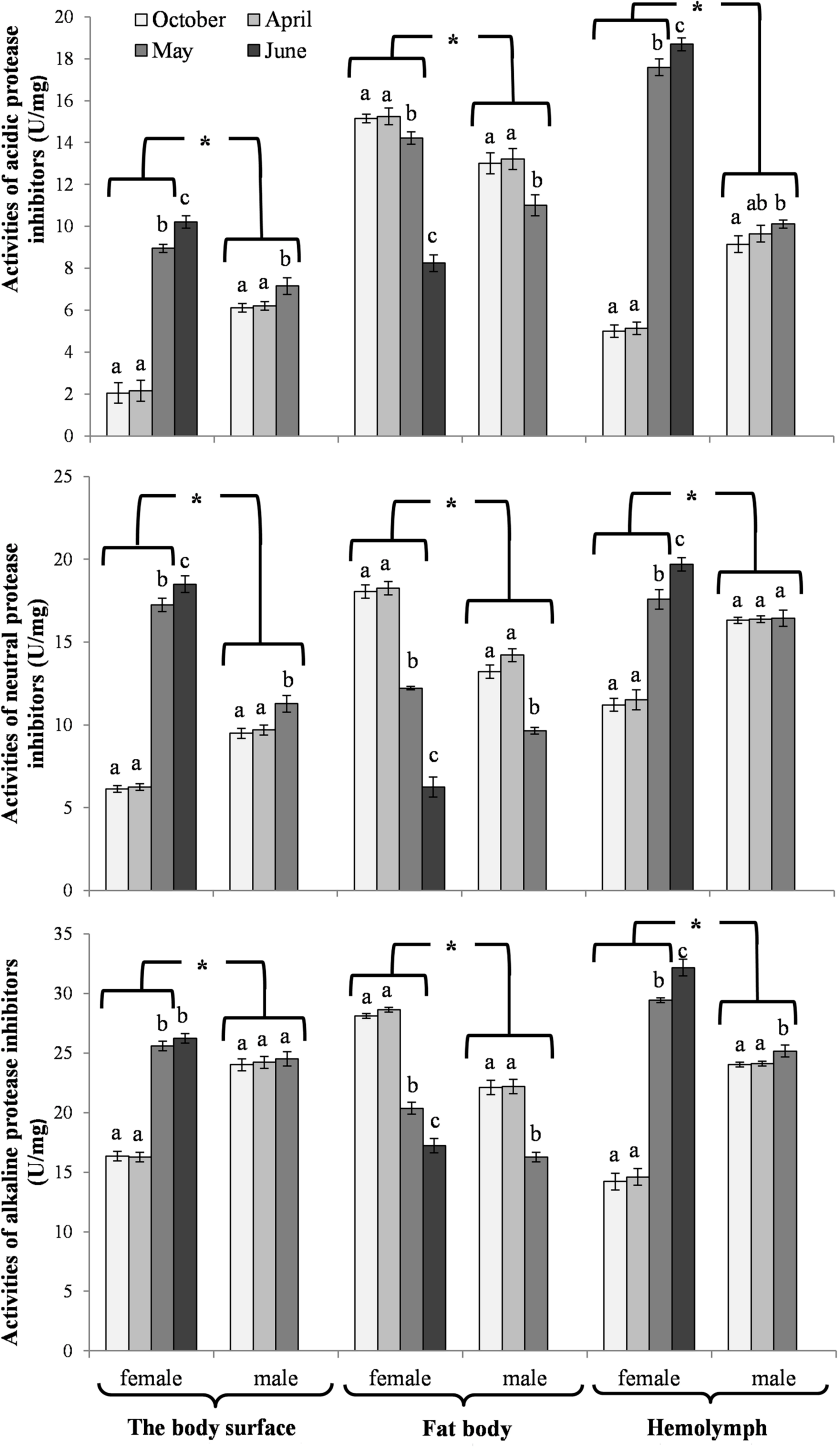
Activities of acidic, neutral and alkaline protease inhibitors on the cuticles and also in the fat bodies and hemolymph in *Osmia rufa*. Various lowercase letters indicate that the month averages differ significantly for comparisons made within each sex and tissue at P ≤ 0.05. The *asterisks* indicate significant differences (P ≤ 0.01) difference between sexes compared for the each month separately. The comparison was made separately within each of the tissue. Males were not found in June.

**Fig 4:**
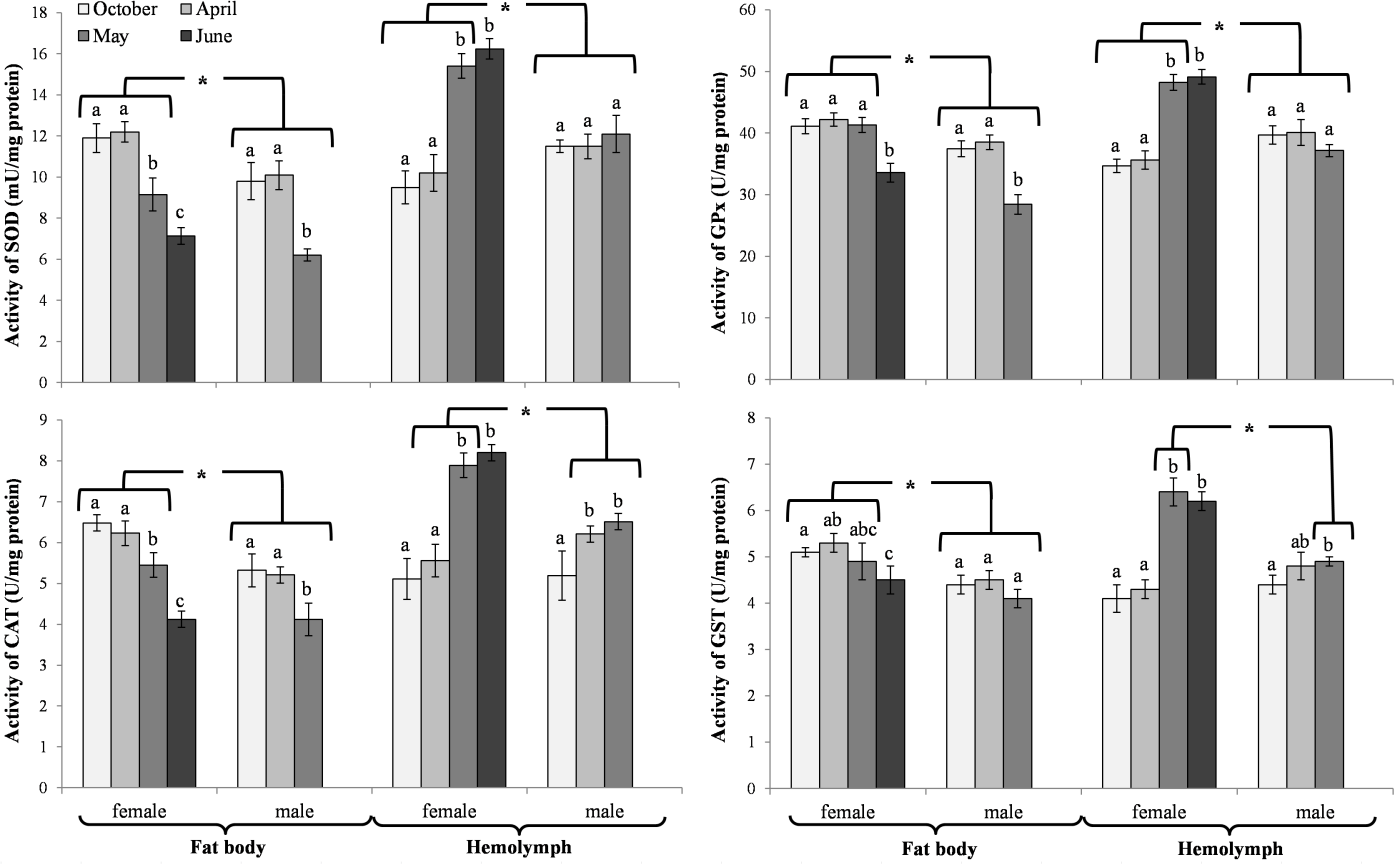
Activities of antioxidant enzymes in the fat bodies and hemolymph in *Osmia rufa*. Various lowercase letters indicate that the month averages differ significantly for comparisons made within each sex and tissue at P ≤ 0.05 The *asterisks* indicate significant differences (P ≤ 0.01) difference between sexes compared for the each month separately. The comparison was made separately within each of the tissue. Males were not found in June.

**Fig 5:**
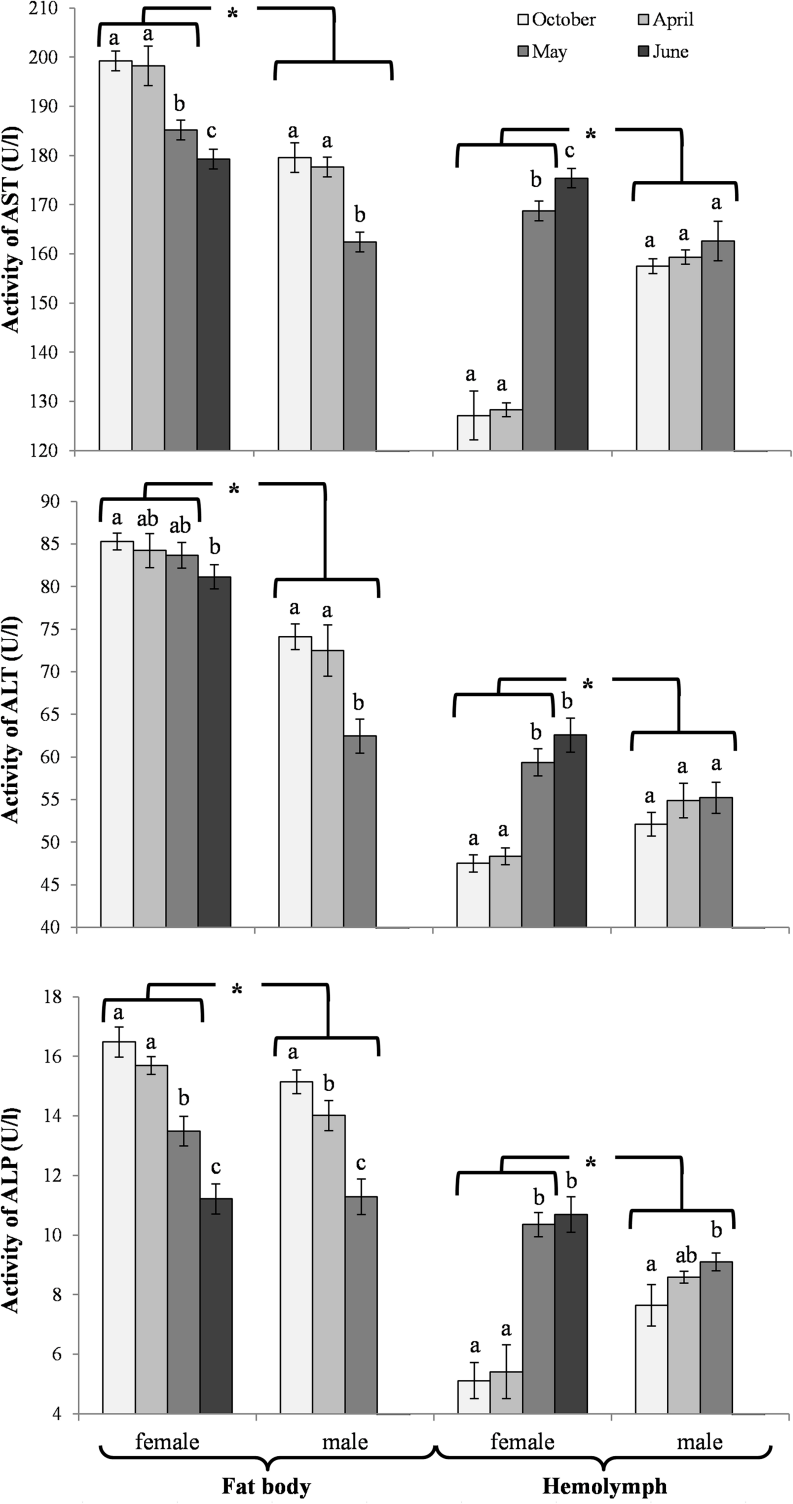
Activities of aspartate aminotransferase (AST), alanine aminotransferase (ALT) and alkaline phosphatase (ALP) in the fat bodies and hemolymph in *Osmia rufa*. Various lowercase letters indicate that the month averages differ significantly for comparisons made within each sex and tissue at P ≤ 0.05. The *asterisks* indicate significant differences (P ≤ 0.01) difference between sexes compared for the each month separately. The comparison was made separately within each of the tissue. Males were not found in June.

**Fig 6:**
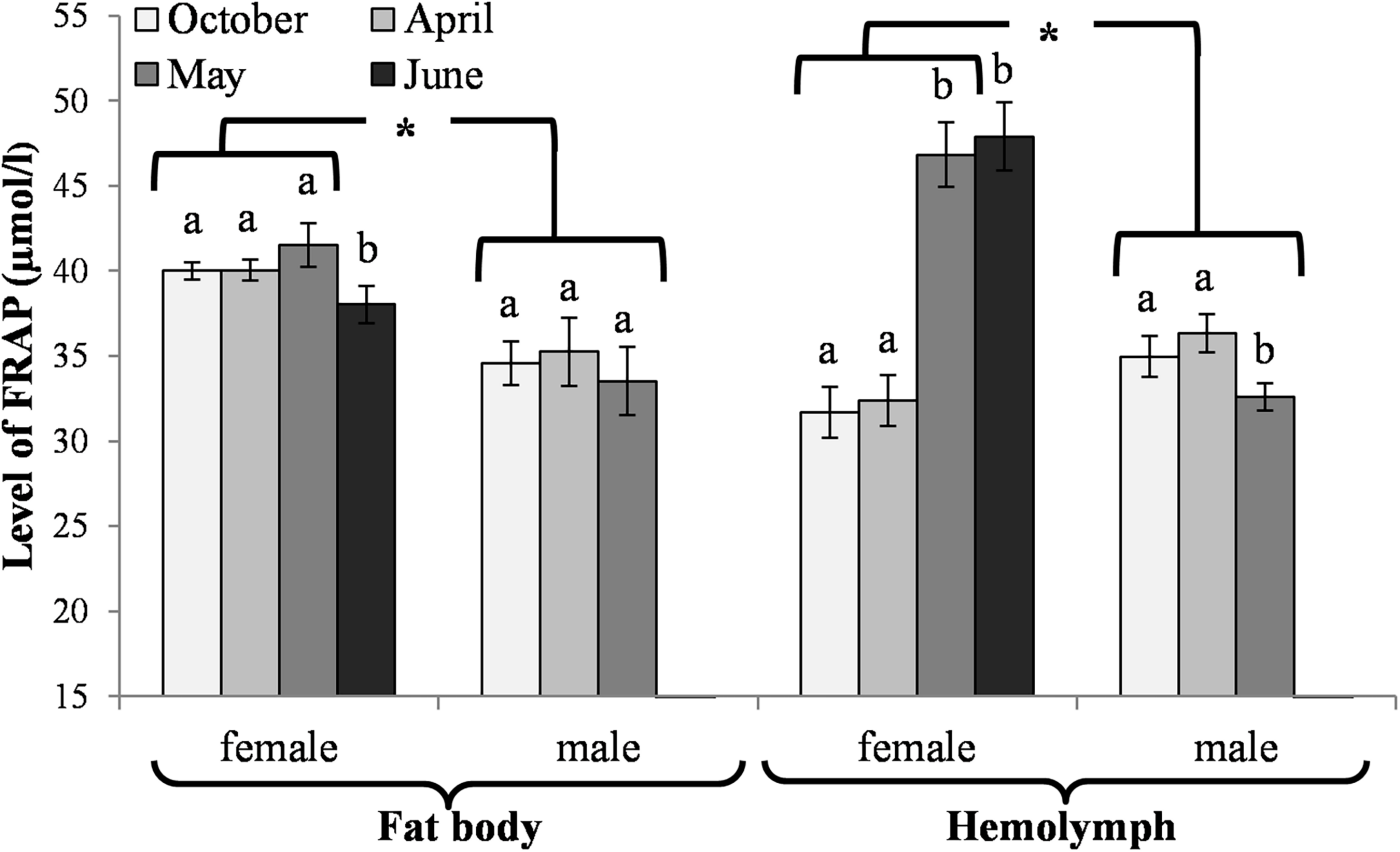
Levels of the total antioxidant potential (FRAP) in the fat bodies and hemolymph in *Osmia rufa*. Various lowercase letters indicate that the month averages differ significantly for comparisons made within each sex and tissue at P ≤ 0.05. The *asterisks* indicate significant differences (P ≤ 0.01) difference between sexes compared for the each month separately. The comparison was made separately within each of the tissue. Males were not found in June.

**Fig 7:**
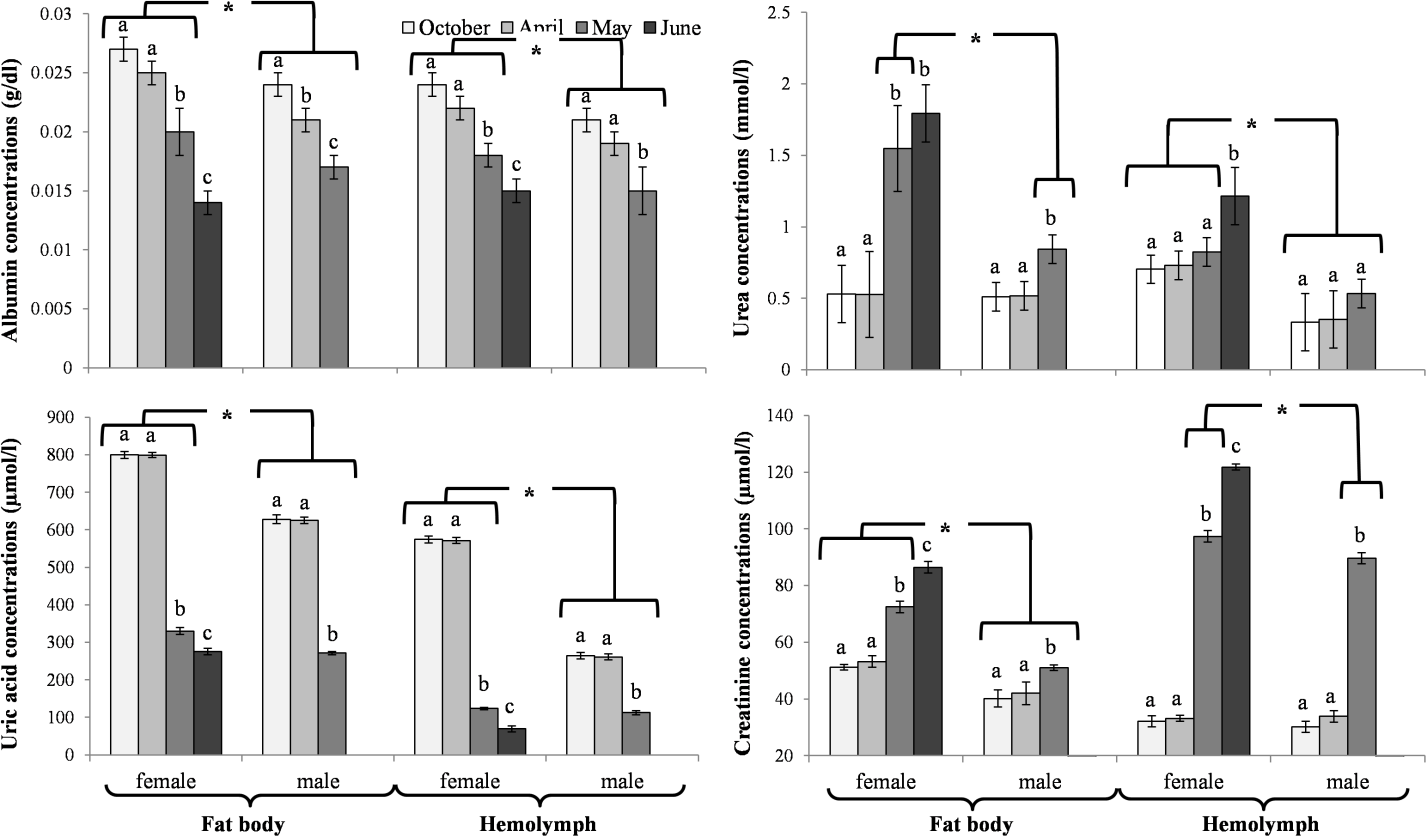
Concentrations of non-enzymatic antioxidants in the fat bodies and hemolymph in *Osmia rufa*. Various lowercase letters indicate that the month averages differ significantly for comparisons made within each sex and tissue at P ≤ 0.05. The *asterisks* indicate significant differences (P ≤ 0.01) difference between sexes compared for the each month separately. The comparison was made separately within each of the tissue. Males were not found in June.

**Fig 8:**
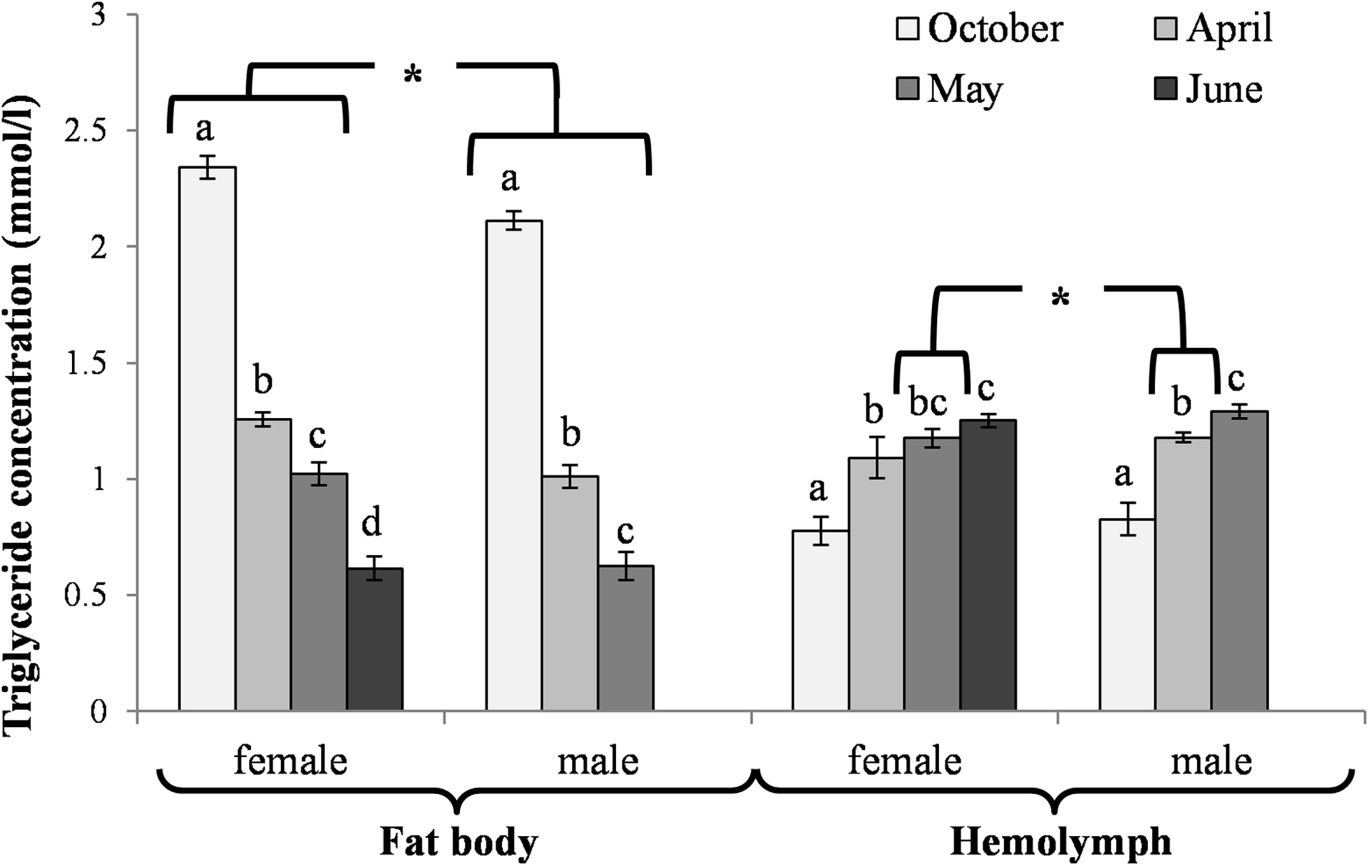
Triglyceride concentration in the fat bodies and hemolymph in *Osmia rufa*. Various lowercase letters indicate that the month averages differ significantly for comparisons made within each sex and tissue at P ≤ 0.05. The *asterisks* indicate significant differences (P ≤ 0.01) difference between sexes compared for the each month separately. The comparison was made separately within each of the tissue. Males were not found in June.

**Fig 9:**
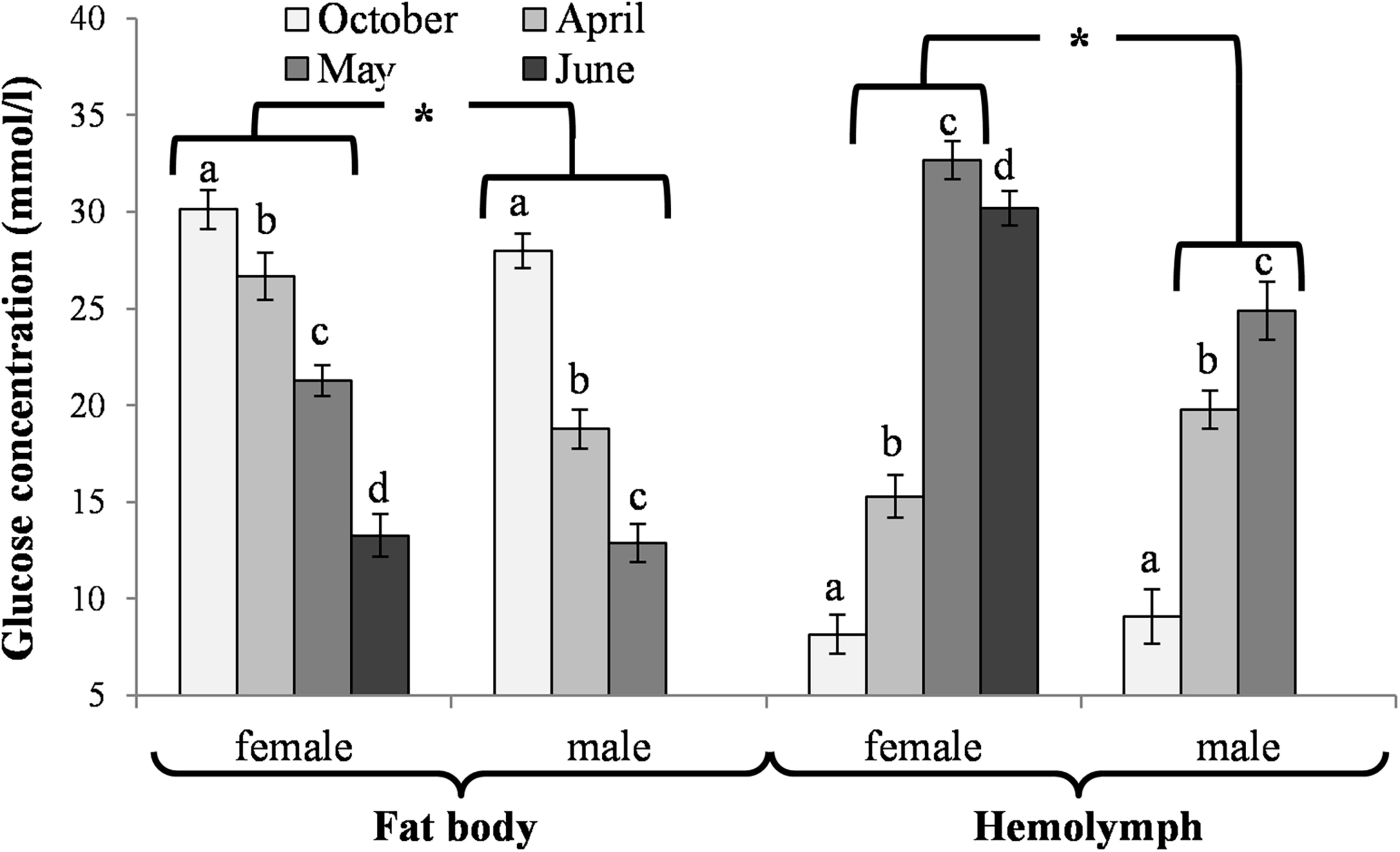
Glucose concentration in the fat bodies and hemolymph of *Osmia rufa*. Various lowercase letters indicate that the month averages differ significantly for comparisons made within each sex and tissue separately at P ≤ 0.05. The *asterisks* indicate significant differences (P ≤ 0.01) difference between sexes compared for the each month separately. The comparison was made separately within each of the tissue. Males were not found in June.

**Fig 10:**
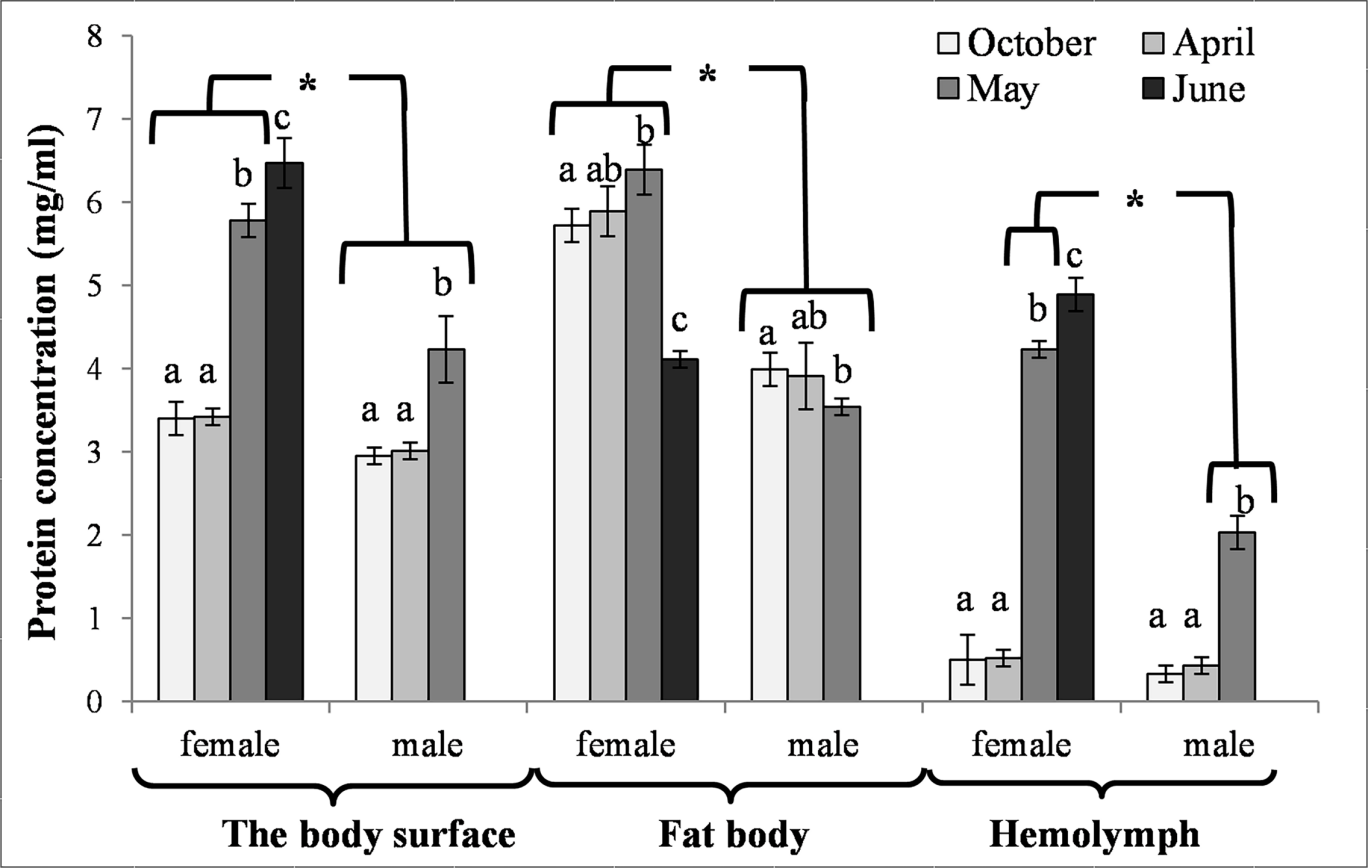
Cuticle, fat body and hemolymph protein concentrations in *Osmia rufa*. Various lowercase letters indicate that the month averages differ significantly for comparisons made within each sex and tissue at P ≤ 0.05. The *asterisks* indicate significant differences (P ≤ 0.01) difference between sexes compared for the each month separately. The comparison was made separately within each of the tissue. Males were not found in June.

**Fig 11:**
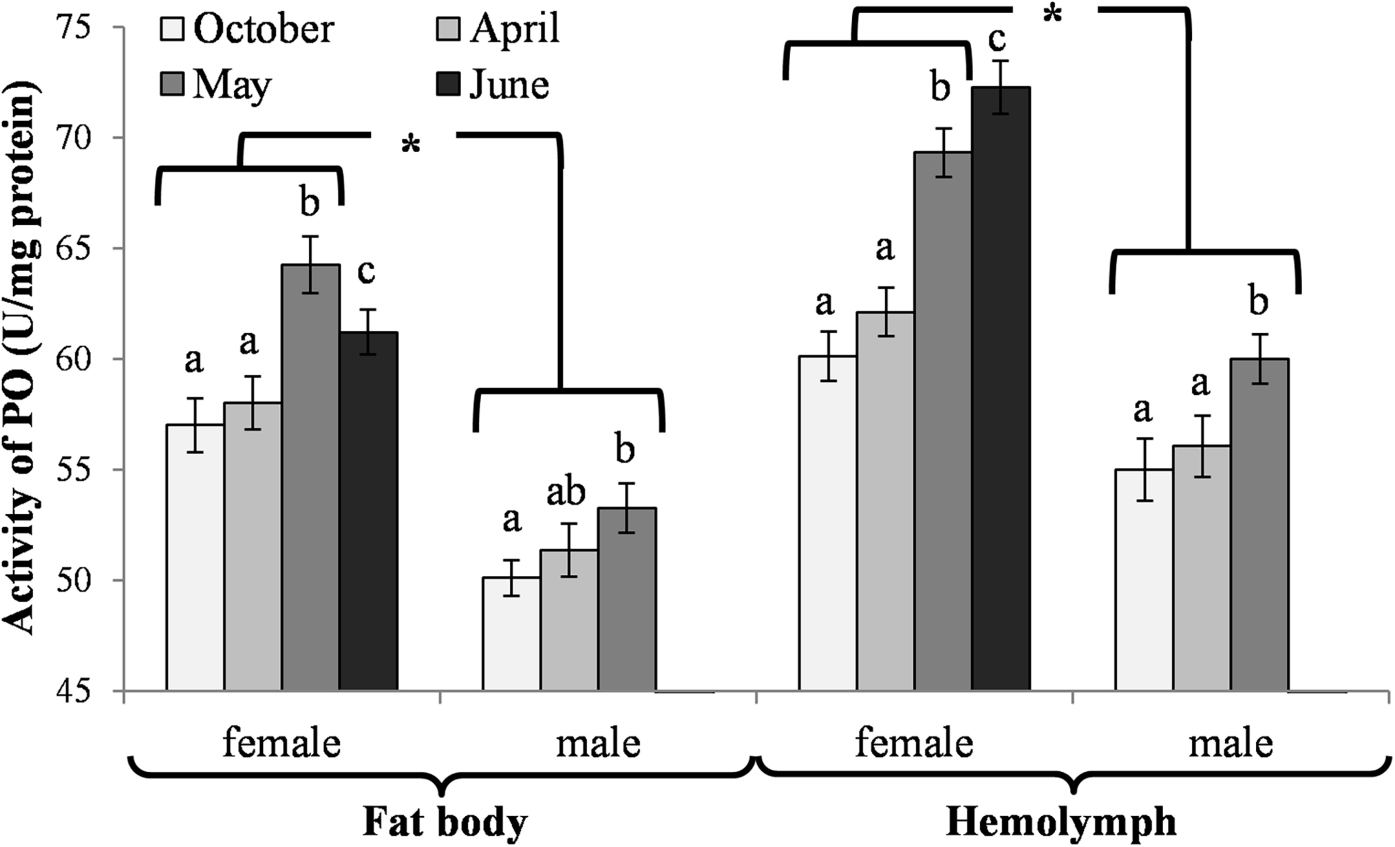
Phenoloxidase (PO) activities in the fat bodies and hemolymph in *Osmia rufa*. Various lowercase letters indicate that the month averages differ significantly for comparisons made within each sex and tissue at P ≤ 0.05. The *asterisks* indicate significant differences (P ≤ 0.01) difference between sexes compared for the each month separately. The comparison was made separately within each of the tissue. Males were not found in June.

The activities of acidic, neutral and alkaline proteases (Fig 2) and their inhibitors (Fig 3), antioxidant enzymes (Fig 4) and enzymatic biomarkers (Fig 5), the levels of the total antioxidant potential (FRAP, Fig 6), concentrations of albumin and uric acid (Fig 7), and triglycerides (Fig 8) and glucose (Fig 9) decreased with age in the fat bodies in both sexes of *O*. *rufa*. Similar tendencies were also observed in the case of protein concentrations (Fig 10) in the males. An increase in phenoloxidase (PO) activities (Fig 11) in the males and females and protein concentrations (Fig 10) in the females were observed until May and afterwards, in June, they decreased in the females.

### The body-surface proteolytic system

Protein concentrations and acidic, neutral and alkaline protease activities were higher in the females than in the males (Figs 2 and 10). Similar tendencies were observed in the case of the activities of acidic, neutral and alkaline protease inhibitors in May (Fig 3). The concentrations of proteins (Fig 10) and the activities of alkaline proteases (Fig 2) in the females, and the activities of protease inhibitors (Fig 3) in both sexes increased with the age of the insects. A decrease of neutral protease activities was observed in the males in the process of ageing, whereas the protein concentrations and alkaline protease activities increased. Acidic and neutral protease activities increased in the females until May and subsequently decreased (Fig 2).

### Biochemical changes in the hemolymph

An increase in the activities of protease inhibitors (Fig 3), PO (Fig 11) and enzymatic biomarkers (Fig 5) and the concentrations of urea, creatinine (Fig 7), triglycerides (Fig 8) and glucose (Fig 9) was observed in the hemolymph of both sexes of *O*. *rufa* as the bees progressed in age. Similar tendencies were observed in the case of protein concentrations (Fig 10), FRAP levels (Fig 6) and the activities of alkaline proteases (Fig 2), SOD, GPx and CAT (Fig 4) in the females. On the other hand, a decrease in albumin and uric acid (Fig 7) concentrations was observed in both sexes, as well as a reduction in FRAP levels (Fig 6) and GPx activities (Fig 4), but only in the males. The activities of acidic and alkaline proteases (Fig 2) and GST (Fig 4) increased both in the males and females between October and May, whereas the activities of neutral proteases (Fig 2) increased only in the females.

### General tendencies

The concentrations/activities of the compounds were usually lower in the males than the females (Figs 2–11), independently of the age. Prior to the diapause/overwintering (October), the concentrations/activities of all the compounds were higher in the fat bodies than in the hemolymph. In late spring and in the summer, they increased in the hemolymph and on the body surface, while decreasing in the fat bodies.

### Global DNA methylation levels

**Fig 12:**
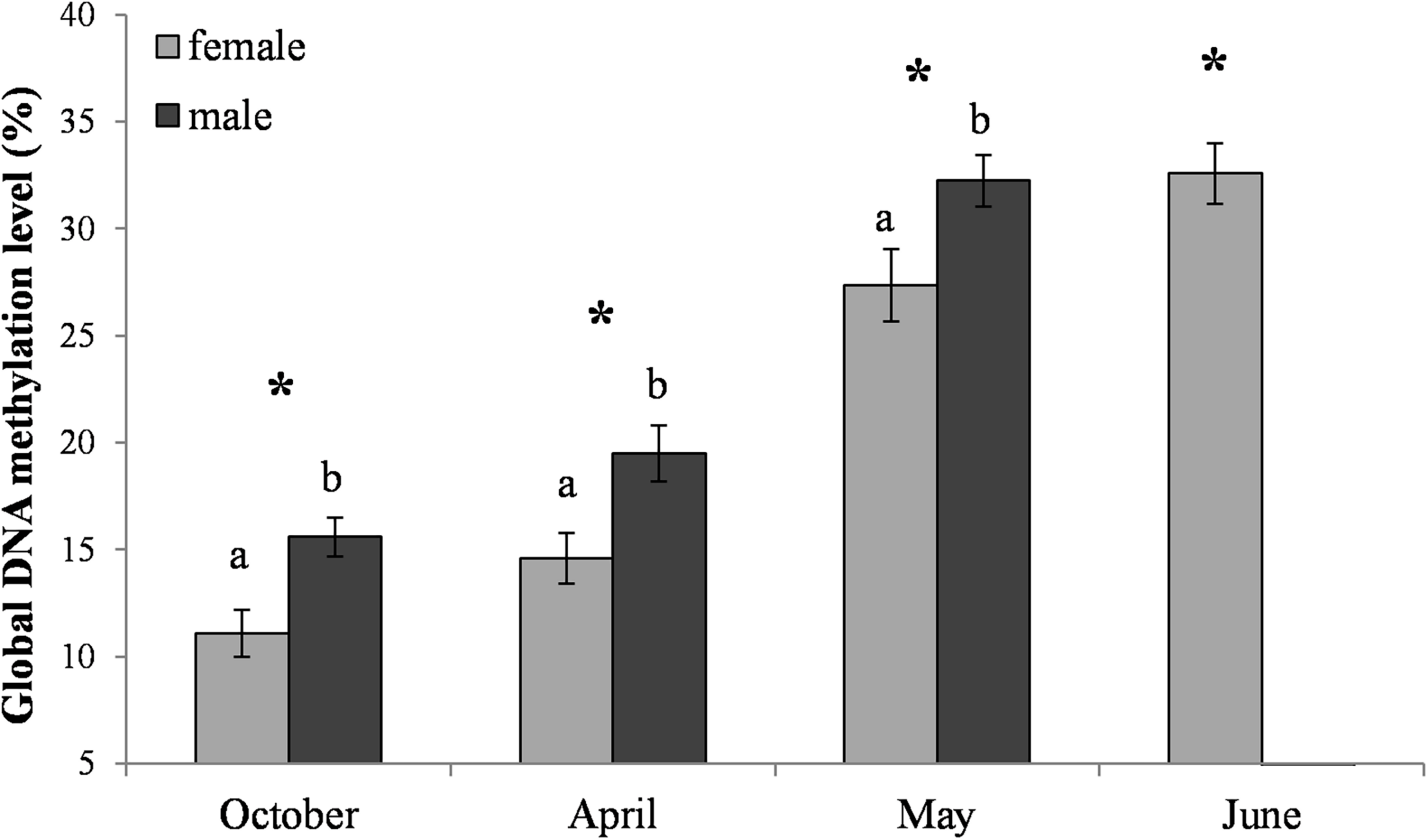
Average global DNA methylation levels (%) in *Osmia rufa*. Various lowercase letters indicate that the month averages differ significantly for comparisons made within each sex separately at P ≤ 0.05. The *asterisks* indicate significant differences (P ≤ 0.01) between the sexes made within each month separately. Males were not found in June.

The global DNA methylation levels increased with age (Fig 12). Higher levels were always observed in the males than in the females. However, the increase was slower in the females than in the males, and the percentages of DNA 5-methylcytosine in the females in June were similar to those observed in the males in May. It is worth noting that the males lived shorter than the females.

## Discussion

### The energy reserves and the fat body tissue patterns

It is believed that the insect fat body is the central storage depot for excess nutrients [46]. In social western honeybees, it is mainly involved in the hormonal control of the storage and mobilization of energy reserves that are crucial for the reproductive anabolic processes and, above all, for the group overwintering capabilities [46,47]. Investigating the diapausing solitary bees, we observed that trophocytes were the only cells that were visible at the beginning of the diapause/overwintering (Fig 1). Their sizes, as well as the numbers of the lipid droplets were the greatest at the beginning of the diapause/overwintering (in October). Afterwards, they decreased with age but these changes were slow until April. Next, they drastically accelerated in May and June in mature flying bees. The changes seem to correspond with the decreasing weights of the entire bodies and the consequent consumption of the fat-body fat reserves during the diapause/overwintering period revealed by Bosch et al. [48], Fliszkiewicz et al. [49] and Sgolastra et al. [50]. Moreover, the highest concentrations of triglycerides (Fig 8) and glucose (Fig 9), i.e. the compounds which are stored as energy reserves (cf. [51]), were observed in October in the fat body cells. Their concentrations systematically decreased in fat body cells but increased in the hemolymph with age from October to June (including the diapause). Triglycerides are used in β-oxidation not only as diapause reserves but also as precursors in the synthesis of eicosanoids and pheromones, and/or as fuel during prolonged periods of flight in spring and summer [46]. Glucose, in turn, is used in the synthesis of chitin, a major cuticle component, and in the synthesis of sugar alcohols, which are needed for adaptation to cold or drought [46,51]. It is also used in lipogenesis and increases the potential of the fat bodies, which explains the higher content of lipids as compared to glycogen in the insect fat bodies, as shown by Zhou et al. [52]. Thus, despite the period of diapause, the *O*. *rufa* fat bodies, although less developed then in *A*. *mellifera* (cf. [46]), are still essential as energy reserves.

### Biochemical defence–the fat body and hemolymph tissues

Our paper is the first to reveal that, unlike in the case of the energy reserves (Figs 8 and 9) and histological features (Fig 1), the levels of the biochemical defence parameters, such as the activities/concentrations of antioxidants, proteases, protease inhibitors, PO and the activities of some crucial enzymatic and non-enzymatic biomarkers were at constant levels in the fat bodies and hemolymph of *O*. *rufa* at the beginning and at the end of the diapause stage (between October and April). All the parameters, as well as the fat body tissue patterns, were dependent on the age and sex of the *O*. *rufa* individuals (Figs 2–11), but only in the mature flying bees. Moreover, oenocytes in the fat bodies changed shape from oval to polygonal and developed yellow granularities in the cytoplasm of *O*. *rufa* along with senescence processes, in May and June. Cruz-Landim [39] and Paes de Oliveira and Cruz-Landim [31] confirmed that it was these proteins and other compounds, including antioxidants and proteases, that were synthetized in these specific cells.

#### Biochemical defence–antioxidative potential

Invariable activities of the antioxidant enzymes during the diapause stage (Fig 4) may be related to inhibited metabolism [48,51]. Dmochowska-Ślęzak et al. [8] obtained similar results but only for homogenates prepared from the entire bodies of the *O*. *rufa* bee in diapause. Our research showed that after the termination of diapause, the activities of SOD, CAT, GST and GPx decreased in the fat bodies and increased in the hemolymph, and the changes depended on the age/senescence in both sexes of *O*. *rufa*, which was observed for the first time. We believe that the bees respond to novel stress stimuli when they leave a nest environment and start “a new life”, exposed to more intensive and varied factors of the “outdoor” environment. It is important information in the light of the growing dependence of agriculture on chemical technologies and increasing environmental pollution that have devastating impacts on bee populations [53]. In particular, free radicals are generated in response to this environmental pressure, including pathogens, pesticides, ultraviolet radiation, ultrasound waves and toxic substances (cf. [8,54]).

It is important to answer the question how the sex of the solitary bees influences biochemical and histological parameters of their biochemical defence. Our results show that the activities of antioxidant enzymes were always higher in the fat bodies of the females in comparison with the males, which was also observed in the bee hemolymph in May (Fig 4). A similar tendency was observed by Dmochowska-Ślęzak et al. [27] in *O*. *rufa* and by Frõhlich et al. [55] in *Megachile rotundata*, but only in the pre-pupae entire-body homogenates.

Similarly to *A*. *mellifera* [21,27,29], solitary bees should be also protected by non-enzymatic antioxidants. Our study showed that, in a similar way to enzymatic antioxidant activities, their concentrations were mostly at constant levels in the fat bodies and in the hemolymph of *O*. *rufa* at the beginning and at the end of the diapause stage (Fig 7). Moreover, it turned out that the albumin and uric acid concentrations decreased whereas the urea and creatinine concentrations increased with age both in the fat body tissues and hemolymph of the mature flying *O*. *rufa* males and females. This was probably due to the increased intensity of processes connected with intercepting reactive oxygen forms and binding ions as a result of the biochemical response to the environmental pressure [56]. This corresponds with the findings of Şapcaliu et al. [57] who observed that concentrations of these compounds increased in honeybees after artificial application of stress factors. It is worth noting that, in a similar way to enzymatic antioxidants, the concentrations of non-enzymatic antioxidants were always higher in the females.

The antioxidant system was supported by high activities of phenoloxidase (PO). Our study showed that, the same as the remaining antioxidants, PO always had higher activities in the *O*. *rufa* females than in the males. It suggests a more effective female antioxidative potential in solitary bees. This also corresponds with Gerloff et al.’s [58] findings that male bumblebees (*Bombus terrestris*) appear to be less immunocompetent than the females. PO retained constant activities at the beginning and at the end of the diapause stage. Subsequently, they also increased with age in mature flying *O*. *rufa* bees (Fig 11). It is quite different than in the case of the bumblebee [59] but similar to *A*. *mellifera* workers [60]. Explaining this phenomenon needs more research because PO acts in a complex way in bee organisms; i.e. by oxidizing tyrosine derivatives to form toxic quinones which are then polymerized into melanin to provide protection from viruses, bacteria, fungi, parasites and parasitoids [61]. During the formation of melanin, toxic metabolites are generated and then degraded/eliminated by the proteolytic and antioxidant systems [62].

#### Biochemical defence–proteolysis

Increased activities of proteases and their inhibitors were observed in the hemolymph in April, when the insects terminated the diapause, particularly in the females (Figs 2 and 3). Zaobidna et al. [16] observed a similar tendency in the extracts of entire *O*. *rufa* bodies for gelatinolytic activities of proteases, whereas Hahn and Denlinger [51] observed increaseed demand for free amino acids–hydrolytic products of proteins–in spring in these bees. It is connected with the need for proteins for egg production in the females [63,64]. However, our study revealed that the proteolytic activities began to decline after the diapause (from April) in the fat bodies of both sexes (Figs 2 and 3) but still remained higher in the females. It may be connected with the reduction of the fat body and down-regulation of many metabolic pathways in this tissue due to the beginning of active flying. Moreover, in the spring, we observed increased activities of the proteolytic systems on the bee cuticle, which is the first insect anti-pathogen barrier [64] protecting against environmental stress and pathogens [20]. Our results revealed that the *O*. *rufa* females had better protection in the cuticle, hemolymph and fat body tissue proteolytic systems than the males. The changes in the activities of the proteolytic systems corresponded with physiological changes in the fat body and hemolymph, particularly with the activities of the antioxidant systems (Figs 4 and 7), PO-related mechanisms (Fig 11), as well as melanization, sclerotization and chitinization [24,25,65], particularly important for biochemical defence and support immunocompetence of solitary bee females.

#### Biochemical defence–enzymatic biomarkers

Enzymatic biomarkers are compounds which support the functions of both the proteolytic and antioxidative systems [21,28]. This study revealed that, similarly to all antioxidant parameters, the activities of aspartate aminotransferase (AST), alanine aminotransferase (ALT) and alkaline phosphatase (ALP) remained at constant levels in the fat bodies and the hemolymph during diapause and afterwards in the process of ageing of the flying insects, and they were usually higher in the females (Fig 5). The age-related tendencies of the biomarker activities in the hemolymph were similar to those described by Strachecka et al. [21,29,66] in *A*. *mellifera* workers.

### Global DNA methylation levels

We confirmed our hypothesis that, in a similar way to the social *A*. *mellifera* [67], the global DNA methylation levels increased with age (also during the diapause) in both sexes of the solitary *O*. *rufa* bees (Fig 12). The increase corresponded with the age-related increase of energy reserves in the hemolymph (Figs 8 and 9) during and after the diapause as well as with the increase in the activities of antioxidant and proteolytic systems along with age in the flying *O*. *rufa* bees (Figs 2–7 and 11). Moreover, we observed higher levels of global DNA methylation in *O*. *rufa* males, which have shorter life-spans, than in the females. In social honeybees, DNA methylation has an important role underlying eusocial characteristics, influencing the developmental divergence between queens and workers, and changing worker behaviour [68]. Moreover, global DNA methylation levels increasing with age in *A*. *mellifera* workers affect task switching [69,70]. In turn, as presented in our study, two periods are important in the life of *O*. *rufa*: the diapause and the active life. A high increase in methylation was observed after the start of flying, especially in the males (Fig 12).

### Evolutionary aspects

Our studies showed that solitary *O*. *rufa* has strong biochemical defences (Figs 2–11). Comparing biochemical defences between solitary and social bees it turned out that solitary bees had higher activities/concentrations of the compounds in the hemolymph than eusocial bees (cf. [21,29,71,72]). It may be connected with higher number of genes and their different expression in solitary and primitively social bees than in *A*. *mellifera* [67,73–78]. From this perspective, our study suggests that, similarly as in the case of immunity, biochemical defence is reduced during evolution. Moreover, the *O*. *rufa* females had better biochemical defences than the males (Figs 2–11). In *A*. *mellifera*, this tendency is opposite and the drones have significantly higher antioxidative capabilities than the queens [79,80]. There are two different views, however, on antioxidant activities in the two female castes of *A*. *mellifera*. According to the first, queens are highly stress-resistant [37]. The second asserts antioxidant activities are lower in the queens than in the workers [81,82]. We compared our study with the results of Parker et al. [83] and Remolina & Hughes [82] and found that the activities of the antioxidative systems increased with age both in the *O*. *rufa* females and social honeybee workers, but they decreased in queens of social bees. With the exception of mating flights, reproductive *A*. *mellifera* females reside among nestmates for their entire life, wherein a court of worker attendants tends to their nutritional and hygienic demands. Therefore, queens appear to be sheltered and not confronted with stressors to the same degree as the other nest inmates [10]. In sharp contrast to social species, solitary bees follow a more risk-sensitive strategy, such as adjusting the sex ratio of their offspring in response to stressors. Even if a species exhibits gregariousness, with hundreds of individual nests in close spatial proximity, the individual females are nevertheless responsible for their own offspring. It appears as if a more diverse immune repertoire may be required by solitary species [9].

In evolution, *O*. *rufa* and *A*. *mellifera* have developed different strategies of wintering. The eusocial *A*. *mellifera* has evolved remarkable abilities to survive extreme seasonal differences in temperature and availability of resources by dividing the worker caste into two groups that differ in physiology and life-span: the summer and winter bees. The physiological adaptation of winter honeybees is associated with an overall decrease in physiological activity, including the down-regulation of the energetically expensive immune system [84] and biochemical defences (Strachecka et. al.’s unpublished data), which results in increased susceptibility to pathogens. The diapause in *O*. *rufa* represents an arrest of development characterized by extremely low metabolic activity. It is an adaptation arising in response to a specific environment. Moreover, the main adaptation in *Osmia* to synchronize adult eclosion with the onset of winter temperatures is to regulate the prepupal summer diapause [85]. In the case of solitary bees, the activities/concentrations of biochemical defence parameters remained at constant levels at the beginning and at the end of the diapause stage, i.e. in October and April (Figs 2–7, 10 and 11); the exceptions were energy reserves (Figs 8 and 9) and histological changes (Fig 1).

Amarasinghe et al. [86] and Sadd et al. [6] suggest that DNA methylation may play an equally important role in the behavioral divergence between solitary and social bees. Comparing the global DNA methylation levels between just emerged (post-diapause) *O*. *rufa* females (Fig 12) and just emerged *A*. *mellifera* queens and workers [45], it turns out that the solitary bee females are more similar to queens than to honeybee workers. It might provide evidence that the ancestors of social bees resembled today’s queens, and it was workers whose phenotype changed more in the course of evolution, whereas today’s queens are more like their ancestors.

## Conclusion

During the diapause, the mechanisms responsible for the energy reserves, related to the morphology of the fat bodies, and the activities of the biochemical defence components functioned differently from one another. After overwintering, the constant levels of these biochemical compounds decreased in the fat bodies and increased in the hemolymph, and they were usually higher in the females. The global DNA methylation level was lower in this sex than in the males. However, the levels increased with age in both *O*. *rufa* sexes.

## Material and methods

Both females and males of *O*. *rufa* were acquired from artificial reed-stalk nests maintained at the apiary of the Laboratory of Environmental Biology and Apidology, University of Life Science in Lublin, Poland (51°15' 50.2" N; 22°31' 38.83" E) in two consecutive years.

### The experimental protocol

In October, part of the reed-stalks were transported from the nests to a conditioned chamber (5°C). The cocoons were shelled, dissected and sexed in order to obtain 200 males and 200 females at the diapause stage for further histological, biochemical and genetic analyses. The remaining stalks were overwintered in the nests, and were subsequently brought out to the conditioned chamber to shell the cocoons in April. 200 males and 200 females, just terminating diapause and beginning the active life stage, were separately acquired from them for the further “April” analysis as described above. The remaining cocoons were carried back to the apiary, placed near new reed-stalk nests within emergency boxes [87]. Consequently, the nests were settled by emerging bees. Next, 200 males and 200 females were separately captured when flying near the nest entrances in May, whereas only 200 females (males were not found) were caught in June for further histological, biochemical and genetic analyses.

The entire procedure produced the following insect base ({[3 months x (200 males + 200 females)] + 1 month 200 females} x 2 years) = 6 x 200 males + 8 x 200 females. Each set of 200 bees, both males and females, was divided into 3 samples. Sample 1., consisting of 40 bees, was used to determine the activities of the cuticular proteolytic system. Sample 2., consisting of 100 bees, was designated to obtain fresh hemolymph. Sample 3., consisting of 60 bees, was used for fat body preparation and to assess the percentages of total DNA methylation.

#### Preliminary processing of the biological material in each of the Sample 1 units

All of the 40 bees from each particular unit in Sample 1 (both female and male samples) were individually refrigerated in 40 sterile Eppendorf tubes (2 ml) at -25^°^C for 1–2 months. Then, each bee was refrozen and individually rinsed for 1 min in 2 ml of distilled water in order to remove impurities. Next, it was shaken/rinsed for 5 min at 3400 rpm in 1 ml of a 1% detergent (Triton X-100) solution in distilled water. Subsequently, the bees were removed and the solutions were filtered through Miracloth and individually refrigerated at -40^°^C for further biochemical analyses. 40 tubes with the frozen solutions, mostly containing cuticle proteins, were obtained from each Sample 1 unit in this way. This provided a total of 240 tubes containing the male solutions and 320 tubes containing the female solutions.

#### Preliminary processing of the biological material in each of the Sample 2 units

A glass capillary (10 μl; the “end to end” type; without anticoagulant; Medlab Products) was individually inserted between the second and third tergite of each of the 100 bees forming a particular Sample 2 unit in order to collect fresh hemolymph. Subsequently, hemolymph volumes were separately measured in each capillary. Hemolymph from all the 10 capillaries (10 bees) was pooled into one sterile Eppendorf tube containing 100 μl of ice-cooled 0.6% NaCl. Ten pooled samples (10 tubes) were obtained in this way. The tubes were immediately refrigerated at -40^°^C for further biochemical analyses. This provided a total of 60 pooled male-hemolymph samples and 80 pooled female-hemolymph samples.

#### Preliminary processing of the biological material in each of the Sample 3 units

Each of the 60 bees forming each particular sample was dissected and the fat bodies were prepared for stereoscopy under a Stereo Zoom Microscope: Olympus SZX16 (Camera: Olympus DP72). The fat bodies from 10 of the 60 bees were placed on glass slides in 0.6% *natrium chloratum* (pro inj.) and covered with cover-glasses. Microscopic preparations were observed and the fat body cells were photographed with Olympus BX61 (Camera: Olympus DP 72; magnification x 40). This provided images of 30 male and 40 female fat bodies. This method enables (undistorted) visualisation of living tissues. The method assumed the use of techniques to maintain the life and normal functioning of organs/tissues (also applied in mammalian transplantology; we successfully put this method in practice, also on other insects). The fat bodies from the remaining 50 of the 60 bees were collected in 10 sterile Eppendorf tubes, 5 bees per each, containing 100 μl of ice-cooled 0.6% NaCl. Next, the tissues were homogenised at 4^°^C and centrifuged for 1 min at 3,000 g. The supernatants (150 male and 200 female supernatants) were immediately refrigerated at -40^°^C for further biochemical analyses. Next, both the heads and thoraces that remained after the processing of each of 60 bees were individually refrigerated in sterile Eppendorf tubes (2 ml) at -25^°^C for 1–2 months. After thawing, the head- and thoracic-DNA was separately extracted from every head/thorax using the DNeasy Blood & Tissue Kit (Qiagen, Germany) following the producer’s instructions. The DNA samples (360 samples of both thorax and head male DNA plus 480 samples of both thorax and head female DNA) were stored at -25^°^C.

### Biochemical analyses of the solutions from the Sample 1 units, the hemolymph from the Sample 2 units and the supernatants from the Sample 3 units

- Protein concentration was determined using the Lowry method, as modified by Schacterle and Pollack [88];
- Proteolytic system activity was determined as follows:
  - activities of acidic, neutral and alkaline proteases according to the Anson method [89] modified by Strachecka et al. [90,91];
  - activities of natural inhibitors of acidic, neutral and alkaline proteases according to the Lee and Lin method [92];
  - proteolytic activities after the addition of pepstatin A, PMSF, iodoacetamide, o-phenantroline (diagnostic inhibitors) according to the Lee and Lin method [92].

### The following additional analyses were performed only in the pooled hemolymph Samples from the Sample 2 units and the supernatants from the Sample 3 units

- The antioxidant system activity was determined as follows:
  - catalase (CAT) activity according to the Aebi method [93] modified by Strachecka et al. [21,29];
  - glutathione peroxidase (GPx) activity according to Chance and Maehly [94] modified by Strachecka et al. [21,29];
  - glutathione S-transferase (GST) activity according to Warholm et al. [95] modified by Strachecka et al. [21,29];
  - superoxide dismutase (SOD) activity according to Podczasy and Wei [96] modified by Strachecka et al. [21,29];
All the activities were calculated per 1 mg of protein.
  - total antioxidant potential (FRAP) according to Benzie and Strain [97];
  - non-enzymatic antioxidant (albumin, uric acid, urea, creatinine) concentrations according to the colorimetric method using monotests from Cormay (Lublin, Poland).
- Phenoloxidase activity was determined using Zufelato et al.’s [98] and Ptaszyńska et al.’s [99] methods.
- Biomarker activity/concentration was determined as follows:
  - activities of aspartate aminotransferase (AST), alanine aminotransferase (ALT) and alkaline phosphatase (ALP) using monotests from Cormay (Lublin, Poland) according to the manufacturer’s procedure.

### Analyses of the DNA from the apian thoraces and heads (Sample 3 units)

The global DNA methylation analyses were performed using the Imprint Methylated DNA Quantification Kit MDQ1 (Sigma, USA) based on the ELISA principle and following the procedure instructions.

### Statistical analysis

Multivariate GLM was carried out (factors: sex, month and year). The impact of the year proved to be insignificant and it has not been further considered. Therefore, only the sex and month impacts were next compared, using two-way ANOVA and Tukey’s test (SAS Institute Version 9.13., 2002–2003 license 86636). Bliss transformation (y = arc sin [x/100]^0.5^) was used to process the percentages of DNA 5-methylocytosine.
